# Effect of red meat, vegetable, tobacco, and alcohol consumption on national cancer mortality index: Data from 1989 to 2013 in 37 developed countries

**DOI:** 10.3389/fnut.2022.929553

**Published:** 2022-06-29

**Authors:** Myung-Bae Park

**Affiliations:** Department of Health and Welfare, Pai Chai University, Daejeon, South Korea

**Keywords:** red meat, vegetable, tobacco, alcohol, cancer, mortality, colon cancer

## Abstract

This study aimed to examine the association between red meat (RM) and death from all types of cancer, as well as its association with the incidence of colon cancer in developed countries. We selected RM, vegetable, tobacco, alcohol consumption, and socioeconomic status as the dependent variables' risk factors and performed ordinary least squares (OLS) and a fixed-effect model (FEM) analysis. Data from 1989 to 2013 for 37 Organization for Economic Cooperation and Development (OECD) countries. According to the FEM, cancer death had statistically significant associations with education level (Coef = −0.022, P = 0.009), total health expenditure (Coef = −0.049, P = 0.000), aging rate (Coef = −0.178, P = 0.000), tobacco consumption (Coef = 0.096, P = 0.000), RM consumption (Coef = 0.107, P = 0.000), and vegetable consumption (Coef = −0.034, P = 0.000). A similar trend was also observed in the 3 and 5-year lagged models. RM consumption also demonstrated a significantly positive association with the incidence of colon cancer in the OLS. According to the scatter plots and fitted lines based on the recommended allowance RM consumption, cancer deaths and incidence of colon cancer increased as consumption increased in the excess consumption group. Regarding vegetable consumption, cancer deaths and incidence of colon cancer decreased as consumption increased in the group exceeding the recommended allowance level. RM consumption was found to be higher than the recommended allowance level. RM consumption increased cancer deaths and the incidence of colon cancer. There is justification for public health interventions to limit RM consumption in major developed countries.

## Introduction

### Red meat consumption

From a nutritional perspective, red meat (RM) is rich in essential nutrients, such as proteins, vitamin B, heme iron, and zinc. Furthermore, fatty acids found in lean tissue, such as n_3 polyunsaturated and conjugated linoleic acids, are known for their health benefits (1, 2). Nevertheless, there have been several reports that inappropriate intake of RM is not good for health. Saturated fats found in RM increase levels of low-density lipoprotein (LDL) and cholesterol, which have adverse effects on cardiovascular health (3). Since the 1950s, the American Heart Association has recommended reducing the intake of dietary cholesterol and saturated fats to prevent cardiovascular disease. Current guidelines suggest that saturated fats should account for < 7–8% of the total daily calories, and the consumption of cholesterol should be < 300 mg per day (4). Furthermore, the genotoxicity and oxidative stress from RM consumption can induce the destruction of DNA and adenoma formation, which can lead to cancer (5). RM consumption increases the incidence of colon and rectal cancers and is associated with breast (6), prostate, and pancreatic cancers (7). In 2007, the World Cancer Research Fund (WCRF) recommended < 71 g of daily intake of RM (8), while the International Agency for Research on Cancer (IARC) officially named RM as a group 2A carcinogen in 2015. Particularly, heme iron from RM has been identified as a risk factor for colon carcinogenesis (9).

Overall, although RM is a major food category that provides essential nutrients, excessive consumption of RM has been recognized in modern society as posing negative effects on health, including its association with cancer. However, the effect of over-consumption of RM on cancer from a public health perspective is unclear. This is because most related studies thus far have presented findings at the individual level rather than at the population or national level. However, clinical or individual studies do not lead to population-level health outcomes due to a combination of ecological factors (10). Individual-level studies suggest that people who consume more meat are more likely develop cancer and die. However, they do not conclude that cancer death and incidence are higher in countries with high meat consumption. Therefore, it is not free from an individualistic fallacy to claim that RM should be restricted at the community or country level through studies using individuals as a unit (11, 12). As such, study findings can differ within the same variables, depending on the research methods and scope of analysis. Nevertheless, most findings thus far have been at the individual level (13), and additional research is needed to examine associations at a nationwide level. A study by Ranabhat. et al. examined the association between RM consumption and life expectancy in 164 countries. The findings demonstrated a positive association between RM consumption and life expectancy in developing countries but a negative association with high-income countries (11). Nonetheless, the limitation that life expectancy can be affected by many factors other than RM was discussed, suggesting the need for additional analysis of health indicators such as cancer, which are more closely related to RM, as a dependent variable.

### Vegetable, tobacco, and alcohol consumption

Vegetables are known to reduce cancer, stroke, heart disease, cataracts, and hypertension, and have a positive effect on health outcomes (12, 14). Vegetable intake is considered an important aspect of cancer prevention through diet, especially that of colon cancer (15). Tobacco smoke, the most common cause of death, is a Group 1 carcinogen that causes 7 million premature deaths each year worldwide (16). Mortality from smoking alone is greater than the combined effect of all other causes, including alcohol consumption, traffic accidents, and acquired immune deficiency syndrome (AIDS) (17). While the effect of consumption of small amounts of alcohol on health is debatable, with regards to cancer, alcohol is known to increase the risk of liver, prostate, and several other types of cancers (18–20).

### Aims and goals

This study aimed to examine the association between RM and death from all types of cancer, as well as the incidence of colon cancer in Organization for Economic Cooperation and Development (OECD) countries between 1989 and 2013. For this, diet-related behavioral variables of vegetable, tobacco, and alcohol consumption and socioeconomic status (SES) were set as control variables.

## Methods and materials

### Subject and data

Our study subjects were OECD countries. Statistics of OECD member countries are relatively well established. Therefore, to compare statistics for policymaking, member states are required to regularly submit statistics in various fields, such as economy, society, and health. In this context, the OECD publishes annual reports in each field, including health at a glance.

The Food Agriculture Organization (FAO) provides RM and other food-related statistics. We used data from 1989 to 2013 from 37 countries that had joined the OECD. In this study, data on RM and vegetable consumption were collected from FAO STAT (http://www.fao.org/faostat), and other data provided by OECD STAT (https://stats.oecd.org/ and https://data.oecd.org/) were used. Both OECD and FAO data are provided by country and year.

### Dependent variables

In this study, deaths due to all types of cancer were included. The number of cancer deaths per 100,000 people was selected as the main dependent variable. Among cancers, colon cancer has already been proven to be closely related to RM. In the case of colon cancer in the OECD STAT, only the incidence can be downloaded. Therefore, we selected the incidence rate of colon cancer as the dependent variable. In the OECD, cancer-related statistics are officially reported by the health ministries of each country, and cancer death is noted based on ICD-10 (C00-C97) and colon cancer based on the diagnosis of C18. The unit was the incidence per 100,000 people.

### Explanatory variables

In FAO STAT, total meat consists of meat obtained from bovine animals, aquatic mammals, mutton, goat, pig, and poultry. Except for poultry, the rest were defined as RM (21). Total vegetable consumption was the sum of onions, peas, potatoes, roots, tomatoes, and others. RM and vegetable consumption were measured in grams per capita day. Tobacco consumption in grams per capita year and alcohol consumption in liters per capita year; both variables are at age ≥15 years.

Consumption is the starting stock plus imports and production, minus exports, seeds, animal feed, disposal, and other non-food uses and ending stock. FAO defines this as food available for consumption (22).


$$\begin{array}{l} Food\;available\;for\;consumption=starting\;stocks+(quantity\;imported+quantity\;produced)-(quantity\;exported+seed\\ \;+animal\;feed+waste+other\;non\text{-}food\;uses)-ending\;stocks \end{array}$$

SES is closely related to cancer (23, 24) and is the factor that most affects health outcomes at the national level (25). The most widely used indicator at the national level is gross domestic product (GDP) per capita (USD). Health expenditure is known to positively affect population health, such as longevity and child mortality (25, 26). Education level is highly correlated with health (27), and we used the percentage of tertiary education completed in the 25–64-year-old population. Total health expenditure (THE) is a concept that encompasses investment costs of goods and health services, administration, and health, including medical treatment services, such as treatment, rehabilitation, and long-term care, and is widely used as a factor that determines health at a macroscopic level. We used THE per capita (USD). Finally, we selected the percentage of the older people over 65 years (aging rate), which affects the overall socioeconomic factors (28).

### Data analysis

A descriptive analysis was conducted on the data pertaining to 24 years in 37 countries for all variables. Furthermore, some variables had missing values, and replacing them improved the predictive power of the model when analyzing panel data (29, 30). As such, multiple imputations using the Markov chain Monte Carlo were performed. Although there are no set standards for the proportion of missing data, substitution was not performed when the proportion of missing data was <50% in this study (29, 31). Particularly, substitution was difficult in this study as the proportion of missing data was higher in colon cancer than in other variables, and data from some countries were unavailable. A correlation analysis was conducted with cancer death, the incidence of colon cancer, and RM, vegetable, tobacco, and alcohol consumption. Moreover, pooled ordinary least squares (OLS) and a fixed-effect model (FEM) were used to determine whether independent variables affected dependent variables. FEM is a widely used model for panel data in units of countries. Furthermore, since independent variables are expected to affect cancer with some time lag, an additional analysis was conducted with a lag of 3 and 5 years. However, there were many missing values in the case of colon cancer, so FEM was not performed, and only OLS was analyzed. Lastly, the regression line may not necessarily be straight, and it may affect the dependent variable only when it is at a certain level. Therefore, we divided the recommended daily allowance into groups of 71 grams or less and excess in the RM (18). In the case of vegetables, the average daily consumption of <500 grams and excess (32). And scatter plots and quadratic curves were checked according to this subgroup. In the analysis, the entity is a country, and the time unit is a year. In addition, all variables were converted to natural logarithms.

## Results

### Descriptive statistics

The average number of cancer deaths per 100,000 was 229.5, of which Mexico (138.7) had the lowest and Hungary (320.3) had the highest number of deaths. The average incidence of colon cancer was 29.3 people per 100,000, with Hungary (43.4) having the highest and Mexico (7.3) having the lowest incidence. The average GDP per capita was $24,888, with Luxembourg (58,860.6) having the highest and Colombia (7,924.1) the lowest GDP. The average of education level was 24.0%, with Canada (40.8%) having the highest and Turkey (11.8%) having the lowest rate. The THE averaged $2,086.4, with the USA (5,308.0) having the highest and Turkey (481.6) the lowest THE. The average aging rate was 13.6%, with Italy (18.3%) having the highest and Mexico (5.2%) having the lowest aging rate. The average tobacco consumption per capita was 1,976.3 grams per capita year, with Greece (3,060.6) having the highest and Luxembourg (1,121.) the lowest. The average alcohol consumption was 9.4 liters per capita/year, with France (13.8) having the highest and Turkey (1.5) the lowest. The average RM consumption was 149.1 g/capita/day. Austria had the highest RM consumption at 209.1 g/capita/day, and Turkey (32.6 g/capita/day) had the lowest. The average vegetable consumption was 511.8 g/capita/day. Greece had the highest vegetable consumption at 958.5 g/capita/day, with Mexico having the lowest at 202.9 g/capita/day (Table 1).

**Table 1:** Summary for dependent and explanatory variables of 37 OECD countries, average from 1989 to 2013.

	**Cancer** **(SD)**	**Colon** **(SD)**	**GDP** **(SD)**	**Education** **(SD)**	**THE** **(SD)**	**Aging rate** **(SD)**	**Tobacco** **(SD)**	**Alcohol** **(SD)**	**RM** **(SD)**	**Vegetable** **(SD)**
OECD	229.5 (36.9)	29.3 (8.3)	24,888.1 (13,349.7)	24.0 (9.4)	2,086.4 (1,390.5)	13.6 (3.7)	1,976.3 (588.0)	9.4 (3.1)	149.1 (49.4)	511.8 (170.4)
Australia	221.6 (17.4)	33 (3.6)	30,604.5 (9,595.2)	29.6 (6.8)	2,393.5 (954.1)	12.5 (0.9)	1,402.9 (315.1)	10.3 (0.3)	209.1 (14.7)	446.8 (20.2)
Austria	229.1 (21)	32.7 (2.8)	31,317.2 (9,117.7)	22.6 (5.6)	2,945.6 (1,060.4)	16 (1.1)	1,954.2 (302.2)	13.3 (0.9)	216.6 (21.7)	437.2 (35.3)
Belgium	244.5 (26.7)	34.3 (2.7)	29,355.2 (8,212)	27.2 (5.9)	2,589.9 (1,020.9)	16.5 (0.9)	2,023.4 (339.1)	11 (0.7)	165.3 (13)	622.2 (72.2)
Canada	233.8 (16.8)	35 (2.7)	30,808.8 (8,201.2)	40.8 (8.3)	2,822.2 (937)	12.7 (1.1)	1,437.8 (279.2)	7.9 (0.5)	168.5 (10.5)	589.1 (27.4)
Chile	220.9 (14.9)	24.5 (3.8)	11,502.7 (5,427.4)	21.3 (2.6)	758.2 (431)	7.9 (1.2)	2,188.8 (305.1)	7.3 (0.8)	103.9 (16.5)	448.1 (42.6)
Colombia	172.2 (14.8)	14.1 (2.5)	7,924.1 (2457.7)	18.0 (4.0)	683 (311.3)	5.9 (0.7)	2,277.5 (220.8)	5.4 (1.3)	59.5 (5)	286.5 (29.8)
Czechia	287.8 (29.2)	36.7 (4)	19,222.5 (6,767.4)	12.7 (3.3)	1,230.7 (622.2)	14 (1.2)	2,468.8 (276.7)	11.7 (0.3)	159.7 (17.5)	497 (101.1)
Denmark	275.1 (18.6)	39.2 (3)	30,541.6 (9,365.1)	26.2 (7.4)	2,665.5 (1,038.3)	15.6 (0.9)	1,605.4 (176.3)	11.7 (1.1)	188.5 (41.9)	501 (37.2)
Estonia	243.5 (11)	24 (4.2)	15,020 (7,106.8)	29.5 (6.1)	2,128.5 (2,424)	15.3 (2.1)	2,208.7 (251.2)	10.6 (2.2)	131.3 (31.9)	622.8 (69.8)
Finland	197.9 (16.4)	25.7 (2.5)	28,160.7 (8866.7)	28.1 (8.7)	2,208.9 (923)	15.4 (1.6)	1,090.1 (236.9)	9.2 (0.7)	144.6 (5.6)	435.5 (20.9)
France	231.4 (18.4)	33.3 (2.5)	27,173.6 (7,211.1)	23.8 (5.2)	2,833.5 (972.3)	15.7 (1.1)	1,718.5 (388.4)	13.8 (1.3)	180.9 (10)	511.1 (48.9)
Germany	232.3 (22.3)	33.4 (3.5)	29,887.3 (7,969.5)	23.5 (2.8)	3,096.7 (987.8)	17.5 (2.3)	2,074.3 (298.7)	12.5 (1.1)	215.5 (26.7)	479.6 (16.5)
Greece	207.2 (6.3)	20.8 (4)	21,249 (6,042.6)	18.6 (5.3)	1,696.6 (646.4)	17.1 (2)	3,060.6 (587.7)	9.1 (1)	168.5 (16.4)	958.5 (70.8)
Hungary	320.3 (23.3)	40.3 (3)	14,184 (5,893.6)	17.8 (3.6)	1,150.5 (418.1)	15.2 (1.2)	1,924.1 (257.9)	12.5 (0.9)	111.3 (18.9)	492.5 (59.4)
Iceland	226.2 (18.9)	32.8 (4.1)	31,933.8 (7,949.1)	27.6 (3.9)	2,668.4 (787)	11.6 (0.6)	1,761 (458.7)	6 (1)	147.9 (9.8)	335.6 (40.3)
Ireland	258.6 (19.8)	36.3 (2.1)	31,006.7 (12,499.1)	24.9 (9.4)	2,304.8 (1,288.8)	11.3 (0.3)	2,002.2 (470.5)	12.3 (1.3)	175.3 (14.4)	600.8 (34.4)
Israel	207.7 (15.7)	28.2 (4.3)	24,607.4 (4,031)	33.8 (9.8)	1,463 (529.4)	9.7 (0.4)	1,503.3 (247.8)	2.3 (0.4)	72.6 (14.5)	699.2 (93.8)
Italy	232.1 (19.9)	32.1 (2.2)	27,547.5 (6,175)	13.1 (5.9)	2,137.5 (677.1)	18.3 (2.1)	1,797.2 (262.9)	9 (1.2)	173.2 (5.8)	599.3 (53.4)
Japan	200.7 (11.3)	27.5 (3.8)	28,137.8 (6,028.4)	34.4 (8.5)	2,156.1 (931.3)	18 (4.1)	2,709 (539.1)	8.3 (0.7)	76.8 (5)	388.8 (22.6)
Latvia	243.5 (6.5)	16.6 (7.5)	9,918 (7,556.8)	18.1 (7.3)	1,215 (954.8)	15.4 (2.3)	2,547.2 (484.6)	8.9 (1.5)	106.1 (28.9)	670.4 (56.8)
Lithuania	236.8 (5.6)	20.4 (3)	11,361 (6,000)	23.2 (10.8)	1001.4 (368.7)	12.6 (1.5)	2494.7 (280.7)	11.6 (2.5)	129.5 (19)	673.5 (52)
Luxembourg	235.2 (29.3)	35.5 (2.6)	58,860.6 (2,2375.3)	28.1 (6.2)	3,656.6 (1,364.1)	13.9 (0.2)	1,121.8 (560.6)	13 (1.1)	198.2 (27.4)	468.9 (85.2)
Mexico	138.7 (13.7)	8.6 (2.7)	11,421.9 (3,268.5)	17.0 (4.7)	982.1 (316.1)	5.2 (0.8)	1,831.2 (384.5)	4.8 (0.5)	79.4 (7.2)	202.9 (27.4)
Netherlands	256.6 (16.9)	35.8 (3.3)	32,941.8 (10,349.3)	25.0 (6.6)	2,952.4 (1,237.6)	14.1 (1.2)	2,248.2 (519.5)	9.7 (0.4)	192.3 (25.1)	537 (36.5)
New Zealand	242.5 (20.4)	36.2 (3.6)	23,128.7 (6,648.6)	30.1 (6.6)	1,952.5 (784.3)	12 (0.8)	1,246.4 (338.4)	9.3 (0.4)	201.7 (24.7)	592.4 (95.1)
Norway	223.6 (11.6)	32.4 (4.1)	39,201.1 (16,567.9)	29.8 (7.1)	3,100.9 (1,378)	15.4 (0.6)	1,455 (341.6)	5.7 (0.7)	132.6 (6.4)	425.1 (13.1)
Poland	255.3 (9.5)	26.2 (4.2)	12,623.1 (5,964)	15.7 (4.8)	786.8 (436)	12.3 (1.3)	2,157.4 (366.8)	9.1 (0.9)	156 (14.7)	773.7 (53.1)
Portugal	206.5 (9.6)	25.3 (5)	19,613.6 (5,715.8)	13.8 (4.8)	1,610.2 (678.9)	16.3 (1.9)	2,446.3 (474.9)	12.3 (1.3)	157.9 (16.5)	787.1 (86.5)
Republic of Korea	200.5 (15.7)	20.8 (5.4)	20,550.2 (8,644)	25.9 (10.6)	997 (590.4)	7.9 (2.2)	2,695.9 (486.2)	9 (0.3)	94.7 (24.4)	625.7 (36.7)
Slovakia	264.9 (15.1)	30.1 (3.5)	15,048 (6,905.6)	13.6 (3.1)	1,106.3 (578.1)	11.5 (0.8)	2,337.7 (365.7)	11 (1.1)	135.6 (26.5)	470.9 (67.9)
Slovenia	261 (8.8)	34.6 (3.4)	21,884.8 (5,430)	18.8 (4.8)	1,711.3 (503.2)	14.1 (2.2)	2,068.1 (316.2)	11.7 (1.5)	168.7 (18.2)	414.7 (78)
Spain	213.9 (12.5)	27.4 (2.7)	23,185.4 (7,340.9)	23.4 (7.1)	1,770.9 (730.5)	16 (1.3)	2,089.3 (387.1)	11.3 (1.1)	203.8 (21.2)	697.9 (104.5)
Sweden	203.5 (8.9)	29.3 (2.4)	31,168.3 (9,038.4)	29.5 (5.0)	2,544.4 (1045.4)	17.7 (0.6)	1,521.7 (294.8)	6.6 (0.5)	158 (12.9)	419.1 (27)
Switzerland	208.7 (25.9)	30.3 (3.1)	38,986.1 (10,925.9)	26.9 (5.5)	3,676 (1,168.3)	15.6 (0.9)	2,341 (452.1)	11.1 (1)	162.4 (13.7)	417 (22.7)
Turkey	186.5 (24.5)	15 (4.5)	12,045.9 (4,180.6)	11.0 (3.8)	481.6 (279)	6.2 (1)	2,103 (274)	1.5 (0.1)	32.6 (5.4)	413 (24.5)
U.K	249.5 (19.4)	37.4 (2.3)	27,639 (7,862.1)	28.3 (7.7)	2,151.3 (1,036.8)	16 (0.4)	1,515.7 (460.1)	10.2 (0.7)	138.3 (7.2)	211.7 (9.4)
USA	224.4 (20.6)	34.7 (2.2)	37,455.9 (9,822.1)	35.9 (5.9)	5,308 (2,006.9)	12.7 (0.4)	1,826.8 (272.7)	8.5 (0.3)	196.1 (8.9)	226.7 (13.4)

*The results were calculated after multiple imputations of missing values; SD: standard deviation; Cancer: death from cancer per 100,000; Colon: colon cancer incidence per 100,000; GDP: gross domestic product per capita (US dollars); Education: Percentage of tertiary education completed (25–64 year); THE: total health expenditure per capita (US dollars); Aging rate: rate in a 65+ population; Tobacco: tobacco consumption (grams per capita/year, +15), alcohol consumption (liters per capita/year, +15), RM: red meat consumption (grams per capita/day), Vegetable: vegetable consumption (grams per capita/day). All variables were converted to natural logarithms*.

### Estimates from pearson's correlation, pooled OLS and fixed effect regression

Almost all the variables correlated with each other. The RM consumption was correlated with all variables. Cancer death had a statistically significant correlation with all other variables except THE, and colon cancer was statistically correlated with all variables (Table 2).

**Table 2:** Correlation matrix of cancer death, the incidence of colon cancer, GDP, education, THE, aging rate, tobacco, alcohol, RM, and vegetable consumption (coefficient and *P* value).

	**Cancer**	**Colon**	**GDP**	**Education**	**THE**	**Aging rate**	**Tobacco**	**Alcohol**	**RM**	**Vegetable**
Cancer	1									
Colon	0.592 (0.000)	1								
GDP	−0.076 (0.000)	0.541 (0.000)	1							
Education	0.164 (0.000)	0.369 (0.000)	0.417 (0.000)	1						
THE	−0.059 (0.074)	0.457 (0.000)	0.358 (0.000)	−0.369 (0.000)	1					
Aging rate	0.370 (0.000)	0.566 (0.000)	0.226 (0.000)	−0.410 (0.000)	0.580 (0.000)	1				
Tobacco	0.166 (0.000)	−0.296 (0.000)	−0.317 (0.000)	0.108 (0.000)	−0.455 (0.000)	−0.144 (0.000)	1			
Alcohol	0.488 (0.000)	0.441 (0.000)	0.105 (0.002)	−0.288 (0.000)	0.305 (0.000)	0.556 (0.000)	0.054 (0.101)	1		
RM	0.421 (0.000)	0.566 (0.000)	0.222 (0.000)	−0.197 (0.000)	0.541 (0.000)	0.629 (0.000)	−0.197 (0.000)	0.741 (0.000)	1	
Vegetable	0.290 (0.000)	0.094 (0.004)	−0.088 (0.007)	0.089 (0.007)	−0.135 (0.000)	0.294 (0.000)	0.204 (0.000)	0.198 (0.000)	0.192 (0.000)	1

*Cancer: death from cancer per 100,000; Colon: colon cancer incidence per 100,000; GDP: gross domestic product per capita (US dollars); Education: Percentage of tertiary education completed (25–64 year); THE: total health expenditure per capita (US dollars); Aging rate: rate in a 65+ population; Tobacco: tobacco consumption (grams per capita/year, +15), alcohol consumption (liters per capita/year, +15), RM: red meat consumption (grams per capita/day), Vegetable: vegetable consumption (grams per capita/day). All variables were converted to natural logarithms*.

The pooled OLS data from 1989 to 2013 showed that the cancer death rate was associated with GDP [Coefficient (Coef) = −0.007, *P* = 0.028], THE (Coef = −0.092, *P* = 0.000), aging rate (Coef = 0.159, *P* = 0.000), alcohol consumption (Coef = 0.072, *P* = 0.000), and RM consumption (Coef = 0.123, *P* = 0.000). Colon cancer incidence was associated with GDP (Coef = 0.102, P = 0.000), education level (Coef = 0.081, *P* = 0.003), THE (Coef = −0.066, *P* = 0.001), aging rate (Coef = 0.366, *P* = 0.000), tobacco consumption (Coef = −0.107, *P* = 0.001), and RM consumption (Coef = 0.248, *P* = 0.000) (Table 3).

**Table 3:** Association between cancer death and incidence of colon cancer and each independent variable by pooled OLS analysis, 1989–2013.

	**Cancer death**		**Incidence of colon cancer**	
	**Coef. (T)**	***P*** **value**	**Coef. (T)**	***P*** **value**
GDP	−0.007 (−2.20)	0.028	0.102 (15.49)	0.000
Education	−0.015 (−1.13)	0.257	0.081 (2.97)	0.003
THE	−0.092 (−9.30)	0.000	−0.066 (−2.97)	0.001
Aging rate	0.159 (8.08)	0.000	0.366 (9.20)	0.000
Tobacco	0.015 (1.00)	0.318	−0.107 (−3.49)	0.001
Alcohol	0.072 (4.96)	0.000	0.058 (1.95)	0.051
RM	0.123 (6.78)	0.000	0.248 (6.77)	0.000
Vegetable	0.010 (0.69)	0.489	−0.015 (−0.53)	0.595
F	75.51		148.29	
Adj. R-square	0.394		0.562	
Number of observations	918		918	

*Cancer: death from cancer per 100,000; Colon: colon cancer incidence per 100,000; GDP: gross domestic product per capita (US dollars); Education: Percentage of tertiary education completed (25–64 years); THE: total health expenditure per capita (US dollars); Aging rate: rate in an 65+ population; Tobacco: tobacco consumption (grams per capita/year, +15), alcohol consumption (liters per capita/year, +15), RM: red meat consumption (grams per capita/day), Vegetable: vegetable consumption (grams per capita/day). All variables were converted to natural logarithms*.

Longitudinal analysis was performed using FEM (non-lagged) and 3- and 5-year lagged analyses. Cancer death had statistically significant associations with education level (Coef = −0.022, *P* = 0.009), THE (Coef = −0.049, *P* = 0.000), the aging rate (Coef = −0.178, *P* = 0.000), tobacco consumption (Coef = 0.096, *P* = 0.000), RM consumption (Coef = 0.107, *P* = 0.000), and vegetable consumption (Coef = −0.034, *P* = 0.000). In the 3-year lagged model, education level (Coef = −0.030, P = 0.003), THE (Coef = −0.040, *P* = 0.000), aging rate (Coef = −0.157, *P* = 0.000), tobacco consumption (Coef = 0.094, *P* = 0.000), and RM consumption (Coef = 0.071, *P* = 0.000) were statistically related. In the 5-year lagged model, THE (Coef = −0.043, *P* = 0.000), aging rate (Coef = −0.077, *P* = 0.015), tobacco consumption (Coef = 0.083, *P* = 0.001), alcohol consumption (Coef = 0.068, *P* = 0.001), and RM consumption (Coef = 0.043, *P* = 0.027) were related (Table 4).

**Table 4:** Association between GDP, education, THE, aging rate, RM, vegetable consumption and cancer death by fixed-effect model, 1989–2013.

	**Fixed effect**		**Lagged 3 year**		**Lagged 5 year**	
	**Coef. (T)**	***P*** **value**	**Coef. (T)**	***P*** **value**	**Coef. (T)**	***P*** **value**
GDP	0.001 (0.78)	0.437	−0.000 (−0.03)	0.977	−0.003 (−0.88)	0.380
Education	−0.022 (−2.62)	0.009	−0.030 (−3.00)	0.003	−0.019 (−1.58)	0.115
THE	−0.049 (−9.74)	0.000	−0.040 (−7.13)	0.000	−0.043 (−6.82)	0.000
Aging rate	−0.178 (−8.20)	0.000	−0.157 (−5.78)	0.000	−0.077 (−2.43)	0.015
Tobacco	0.096 (10.34)	0.000	0.094 (9.70)	0.000	0.083 (8.43)	0.000
Alcohol	0.016 (0.94)	0.349	0.034 (1.71)	0.087	0.068 (3.26)	0.001
RM	0.107 (7.40)	0.000	0.071 (3.91)	0.000	0.043 (2.22)	0.027
Vegetable	−0.034 (−1.80)	0.000	−0.014 (−0.53)	0.527	−0.021 (−1.00)	0.315
F		124.89		114.07		117.03
Adj. R-square		0.539		0.432		0.396
Number of observations		918		807		733
Groups		37		37		37

*Cancer: death from cancer per 100,000; Colon: colon cancer incidence per 100,000; GDP: gross domestic product per capita (US dollars); Education: Percentage of tertiary education completed (25-64 year); THE: total health expenditure per capita (US dollars); Aging rate: rate in a 65+ population; Tobacco: tobacco consumption (grams per capita/year, +15), alcohol consumption (liters per capita/year, +15), RM: red meat consumption (grams per capita/day), Vegetable: vegetable consumption (grams per capita/day). All variables were converted to natural logarithms*.

### Scatter plots and fitted lines by sub-group analysis of red meat and vegetable consumption

**Figure 1:**
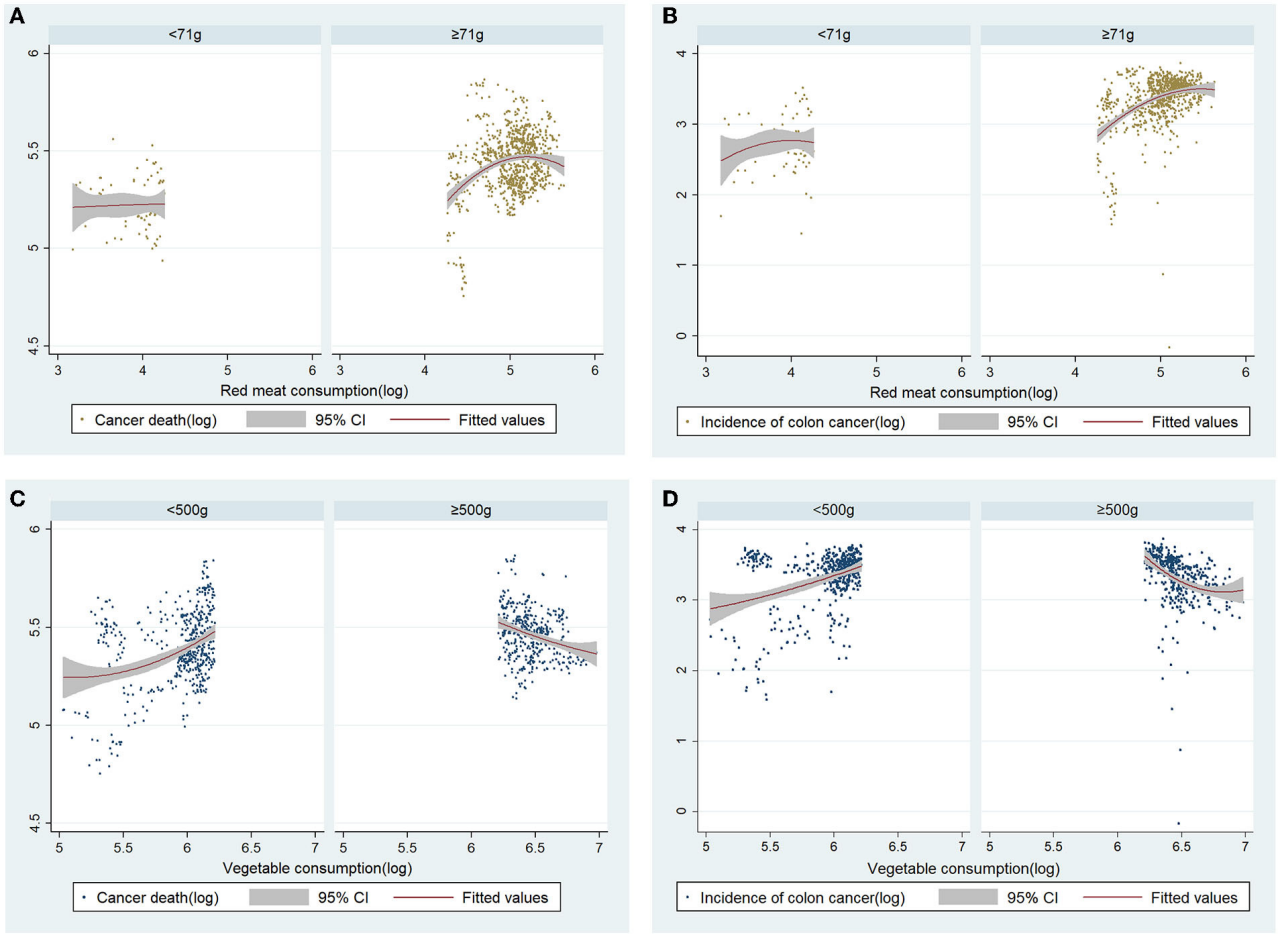
Correlation between red meat consumption, cancer death, and incidence of colon cancer; sub-group analysis by a recommended allowance of red meat a day (71 grams). Scatter plots and quadratic curves for red meat consumption and **(A)** cancer death, and **(B)** incidence of colon cancer. All variables were converted to natural logarithms. Correlation between vegetable consumption, cancer death, and incidence of colon cancer; sub-group analysis by recommended vegetable requirements a day (500 grams). Scatter plots and quadratic curves for vegetable consumption, **(C)** cancer death, and **(D)** incidence of colon cancer. All variables were converted to natural logarithms.

In the case of RM, the slope did not change as consumption increased in the group below the recommended allowance. However, in the excess group, as consumption increased, cancer death also increased, and when it was above a certain level, the slope decreased. In the case of vegetables, the death rate increased as consumption increased in the group below the recommended level. However, the death rate decreased as consumption increased in the group above the recommendation, as was the incidence of colon cancer (Figure 1).

## Discussion

Processed meat is a Group 1 carcinogen that should be avoided. In contrast, RM is considered a limiting food instead of an avoidable food (21). For this reason, whether the consumption of RM should be as restricted as processed meat remains debatable. In general, in developed countries, health problems caused by the overconsumption of meat receive more attention than those caused by a lack of nutrition. In this study, OECD countries were found to have consumed an average of 149 g of RM per day. This is approximately twice the daily recommended intake of 71 g (8), and a consumption rate higher than this was observed in all countries except Turkey.

In OLS, RM was positively associated with cancer death, while non-lagged, 3-year, and 5-year lagged models of FEM showed a positive association with cancer death. Within OECD countries, RM demonstrated a positive association with nationwide cancer death rates, which suggests that RM is a health threat that requires appropriate control for cancer prevention and that public health control for RM has not been well implemented in major developed countries. However, an FEM analysis of colon cancer incidence was not performed in this study due to limitations in data collection. Nevertheless, RM also demonstrated a statistically positive association with the incidence of colon cancer in the pooled OLS analysis.

Moderate meat intake is not yet a matter of concern for increased cancer incidence, and the WCRF recommends consuming no more than 350 to 500 grams of RM per week for cancer prevention (13, 18). Although RM consumption in most countries included in this study exceeded this level, the average consumption was significantly lower than the 200 g per day considered as ‘high consumption′ and only 5 countries had most countries included. Nevertheless, it is clear that RM consumption increases cancer incidence (including colon cancer) and mortality.

We can obtain more information through sub-group analysis according to the consumption of RM. In the case of RM, the positive association between cancer death and incidence of colon cancer was more clearly confirmed in the group exceeding the recommended allowance. Except for a few countries, the recommended allowance was exceeded in many OECD countries. Therefore, to respond more sensitively to cancer prevention policies, it is necessary to implement a nutrition policy that restricts RM consumption in most OECD countries and high RM consuming countries. However, the implications of this study do not involve the restriction of RM consumption in developing countries. This study involved developed countries, and considering that RM remains important from a public health nutrition standpoint following previous research, there will be no need to control consumption in developing countries (11, 33, 34). In this study, vegetable consumption was negatively associated with cancer deaths in FEM, but it was not significant in OLS and lagged models. The association with vegetable consumption could be obtained more clearly through scatter plots through sub-groups. It was confirmed that the dependent variable decreased as the consumption of vegetables increased in the group above the recommended consumption amount.

This is consistent with previous findings that indicate the effectiveness of vegetable consumption above certain levels in reducing cancer incidence (35). However, although vegetable consumption has been demonstrated to reduce the risk of several types of cancer, including colon cancer (36), some studies have demonstrated no association (15, 36). Our findings suggest that a policy intervention to increase consumption of vegetables above the recommended intake may be necessary to prevent cancer, with vegetable consumption in OECD countries averaging 511.8 grams per day, indicating that vegetable consumption is still insufficient in half of the countries. The effects of vegetables on cancer may also vary depending on the type of vegetable consumed (36). However, the type of vegetable is not considered in this study. More interesting findings may be derived from a more curated analysis of the amount of consumption and types of vegetables.

In the FEM and lagged 3 and 5-year model, the number of cancer deaths decreased as the level of education increased. It has been consistently studied that education level has a positive relationship with better health conditions at the individual as well as the national level. Although this mechanism is somewhat complicated, it may be because the higher the education level, the higher the social class or economic level (37) or the education may have affected health by increasing health literacy (27). Meanwhile, the incidence of colon cancer death decreased in both OLS and FEM among SES as the THE increased. THE has been reported to be positively associated with national health level in many studies (25), including a study on OECD countries, where it was deemed to have a negative association with cancer mortality (38, 39). Traditionally, colon cancer has been known as a major health problem in developed countries (40). In the OLS of this study, GDP demonstrated a positive association with the incidence of colon cancer, which is supported by a previous study on 11 Balkan countries (41). Furthermore, a positive association was observed between cancer death and the incidence of colon cancer in the aging rate. Aging is the most well-known cause of cancer, and the aging population is a common cause of increased cancer incidence in developed countries (39).

Smoking is another major risk factor for cancer. Although the incidence of colon cancer demonstrated a negative association with tobacco consumption in the OLS, a positive association was observed in the simple correlation analysis. This may be a problem caused by the lack of data and limitations of the analysis method. Tobacco smoking increases the incidence and mortality rates of colon cancer (42). In most studies thus far, a high level of association was observed between smoking and rectal cancer, but relatively lower or, in some cases, no level of association with colon cancer (42, 43). Furthermore, few studies have assessed this association at the country level. As such, additional research on tobacco consumption and colon and rectal cancer at the national level is needed with supplementary data.

Alcohol consumption was positively associated with cancer death and the incidence of colon cancer in OLS and the lagged 5-year model. Alcoholic beverages have been classified as group 1 carcinogens by the IARC and can cause various types of cancer, including breast, liver, and esophageal cancer (18). Avoiding excessive alcohol consumption is key to cancer prevention, not absolute abstinence (42). Nevertheless, it was clear throughout this study that the level of alcohol consumption in major developed countries contributed to increased cancer incidence and mortality. It is estimated that approximately 4% of the incidence of all cancers worldwide is due to alcohol consumption (44). A previous study stated that even small amounts of alcohol increase the risk of some cancers, and there is no safe level of alcohol consumption (18). Therefore, it is imperative that policies to reduce alcohol consumption be further strengthened for cancer prevention, regardless of the level of consumption.

It is possible to limit excessive intake of RM in terms of individual disease prevention through individual unit research. However, whether these interventions affect health outcomes at the population level is another matter and does not justify a policy to limit consumption at the national level. Our findings indicate that nutritional policies to limit RM consumption may be needed at the national level in OECD countries.

## Limitations and future study

This study is one of the first to confirm the association between RM and national cancer incidence using panel data from 37 countries. Nevertheless, this study had several limitations. First, our research design could not explain the causal relationship between RM and cancer. Second, the findings of this study cannot be generalized at the individual level, as the study was conducted at the country level (ecological fallacy). Likewise, most previous studies have been on individual intake (individualistic fallacy), whereas this study focused on national level consumption, meaning that one must be wary of direct comparisons. Third, results may vary from country to country depending on consumption and food culture. To overcome this limitation, we provided supplementary figures showing the correlation with the dependent variable according to the consumption level by country. This may help understand the relationship between cancer and RM and vegetable consumption in each country. Finally, the reproducibility or reliability of the results is not high for colon cancer due to limitations in the available data. Thus, in the case of colon cancer, data should be sufficiently supplemented and analyzed using more advanced techniques than OLS. Furthermore, setting rectal cancer as a dependent variable will enable more robust research.

## Conclusion

The RM consumption in 37 OECD countries was found to be higher than the recommended intake but lower than the “high consumption” level. The consumption of RM was positively related to deaths due to cancer and the incidence of colon cancer. This finding suggests that an increase in consumption of RM is highly likely to increase cancer death and incidence of colon cancer. Our results justify public health interventions to limit RM consumption in major developed countries. Moreover, the current level of alcohol consumption is likely to contribute to an increase in cancer, and policies to reduce its consumption are necessary. Vegetable consumption was not found to be related to cancer in this study, but consumption above a certain level may effectively prevent cancer.

## Data availability statement

Publicly available datasets were analyzed in this study. The data are available from FAO (http://www.fao.org/faostat) and OECD (https://stats.oecd.org/ and https://data.oecd.org/). If you need the processed data, please contact the author to request the data.

## Author contributions

M-BP initiated the idea and led the formal analysis, reviewed, and edited the final draft of the article.

## Funding

This work was supported by the National Research Foundation of Korea (NRF) grant funded by the Korea Government (MSIT) (NRF-2020R1C1C1007913).

## Conflict of interest

The author declare that the research was conducted in the absence of any commercial or financial relationships that could be construed as a potential conflict of interest.

## Publisher's note

All claims expressed in this article are solely those of the authors and do not necessarily represent those of their affiliated organizations, or those of the publisher, the editors and the reviewers. Any product that may be evaluated in this article, or claim that may be made by its manufacturer, is not guaranteed or endorsed by the publisher.
